# *De Novo* Genome Assembly of Ryegrass Mosaic Virus from a Ryegrass Transcriptome

**DOI:** 10.1128/genomeA.00497-15

**Published:** 2015-05-21

**Authors:** Yeonhwa Jo, Hoseong Choi, Won Kyong Cho

**Affiliations:** Department of Agricultural Biotechnology, College of Agriculture and Life Sciences, Seoul National University, Seoul, Republic of Korea

## Abstract

The ryegrass mosaic virus (RgMV) is a single positive-strand RNA virus belonging to the genus *Rymovirus*. The major natural hosts for RgMV are members of the *Gramineae* species, including ryegrass. Here, we report the nearly complete genome sequence of RgMV by *de novo* assembly using ryegrass transcriptomes.

## GENOME ANNOUNCEMENT

Ryegrass mosaic virus (RgMV), a member of the genus *Rymovirus* in the family *Potyviridae*, is a filamentous virus replicating in the host cytoplasm (1, 2). Its natural hosts are thus very limited to the members of the *Gramineae* species, including *Lolium multiflorum*, *Lolium perenne*, and *Dactylis glomerata* (3, 4). Of these species, perennial ryegrass (*L. perenne*) in the family *Poaceae* is cultivated worldwide and is an important naturalized plant utilized as pasture and forage crops in agriculture (5, 6). Infection by RgMV in *Lolium* spp. and *Dactylis glomerata* exhibits light-green to yellow mosaic symptoms, leading to serious damage to crop yields (2, 7, 8). RgMV is transmitted by both sap and eriophyid mites (*Abacarus hystrix*) (2, 9). RgMV has a monocistronic, positive single-stranded RNA genome encoding a polyprotein and replicates in the cytoplasm (2).

To identify viruses infecting ryegrass, several sets of transcriptome data for *Lolium* species have been screened. Of these species, a recent study provided a reference transcriptome for the perennial ryegrass, which was derived from six different tissues, including the stem, root, meristem, mature leaf, inflorescence, and leaf sheath (10). Six different libraries prepared using a mRNA-Seq sample prep kit (Illumina) were sequenced by the paired-end Illumina GAIIx sequencer. Raw data for all six libraries were downloaded and *de novo* transcriptome assembly was performed for each library using the Trinity program (v.2.0.2) (11). A BLAST search identified three contigs showing strong sequence similarity to the RgMV reference genome sequence (NC_001814.1). Of the three contigs, a contig 9,522 nucleotides (nt) long comprised the nearly complete genome of RgMV. We named the newly identified virus RgMV isolate Denmark (KR061300) according to the identified country. The 5′ and 3′ untranslated region (UTR) sequences of the Denmark RgMV isolate are 16 and 3 nt shorter, respectively, than the reference sequence (NC_001814.1). In general, the 5′ and 3′ UTR sequences for many RNA viruses are highly conserved. The genome of the Denmark RgMV isolate encodes a polyprotein that is processed by proteolytic cleavage into 10 proteins, including PI, HC-Pro, P3, 6K1, C1, 6K2, VPg, NIa, Nib, and Cp. In addition, we confirmed that all six ryegrass samples were infected by RgMV. Thus far, only two genome sequences for RgMV are available. BLASTn results showed 93% and 92% nt sequence identities to the other Danish (NC_001814.1) and Australian (AF035818.1) isolates, respectively. Altogether, our study provides the nearly complete genome sequence for the Denmark RgMV isolate, which is the third genome sequence for RgMV thus far identified. In addition, we demonstrated the successful *de novo* genome assembly of RNA viruses using transcriptome data.

### Nucleotide sequence accession number.

The genome sequence of ryegrass mosaic virus isolate Denmark has been submitted to GenBank (accession no. KR061300).
